# Silencing of Transposable Elements by piRNAs in *Drosophila*: An Evolutionary Perspective

**DOI:** 10.1016/j.gpb.2017.01.006

**Published:** 2017-06-08

**Authors:** Shiqi Luo, Jian Lu

**Affiliations:** 1 State Key Laboratory of Protein and Plant Gene Research, Center for Bioinformatics, College of Life Sciences and Peking-Tsinghua Center for Life Sciences, Peking University, Beijing 100871, China; 2 Beijing Advanced Innovation Center for Food Nutrition and Human Health, College of Food Science and Nutritional Engineering, China Agricultural University, Beijing 100083, China

**Keywords:** Transposable element, piRNA, Hybrid dysgenesis, Evolution, *Drosophila*

## Abstract

**Transposable elements** (TEs) are DNA sequences that can move within the genome. TEs have greatly shaped the genomes, transcriptomes, and proteomes of the host organisms through a variety of mechanisms. However, TEs generally disrupt genes and destabilize the host genomes, which substantially reduce fitness of the host organisms. Understanding the genomic distribution and evolutionary dynamics of TEs will greatly deepen our understanding of the TE-mediated biological processes. Most TE insertions are highly polymorphic in *Drosophila melanogaster*, providing us a good system to investigate the **evolution** of TEs at the population level. Decades of theoretical and experimental studies have well established “transposition-selection” population genetics model, which assumes that the equilibrium between TE replication and purifying selection determines the copy number of TEs in the genome. In the last decade, *P*-element-induced wimpy testis (PIWI)-interacting RNAs (**piRNAs**) were demonstrated to be master repressors of TE activities in ***Drosophila***. The discovery of piRNAs revolutionized our understanding of TE repression, because it reveals that the host organisms have evolved an adaptive mechanism to defend against TE invasion. Tremendous progress has been made to understand the molecular mechanisms by which piRNAs repress active TEs, although many details in this process remain to be further explored. The interaction between piRNAs and TEs well explains the molecular mechanisms underlying **hybrid dysgenesis** for the *I-R* and *P-M* systems in *Drosophila*, which have puzzled evolutionary biologists for decades. The piRNA repression pathway provides us an unparalleled system to study the co-evolutionary process between parasites and host organisms.

## Introduction

Hybrid incompatibility often causes reproductive isolation between two subpopulations, which is important for speciation [1], [2], [3]. The classical genetic mechanism underlying hybrid incompatibility is the Bateson–Dobzhansky–Muller model [4], [5], [6], [7]. Under this model, when one ancestral population is divided into two subpopulations, the original two interacting genes, *aa* and *bb* in the ancestral population, evolve into *AA* and *bb*, and *aa* and *BB* in the two subpopulations, respectively. If the alleles *A* and *B* are incompatible with each other, then the hybrids of the two subpopulations will die or become sterile, resulting in reproductive isolation between these two subpopulations [2]. Among various possible mechanisms that cause hybrid dysgenesis, one important form is intragenomic conflict [4], [5], [6], [7]. Intragenomic conflict arises when genes inside a genome are transmitted by different rules, or when one gene increases its transmission by impairing the host genome. Then the host soon develops strategies to suppress the detrimental effects caused by the selfish genetic elements. The genomic conflicts often lead to hybrid incompatibility or hybrid dysgenesis [4], [5], [6], [7].

Transposable elements (TEs) represent one type of selfish elements in the genomes of almost all species [8], [9], [10], [11], [12], [13], [14], [15], whereas *P*-element-induced wimpy testis (PIWI)-interacting RNAs (piRNAs) represent one type of small RNAs (sRNAs) that repress TEs in animal germlines [16], [17], [18], [19], [20], [21], [22], [23], [24]. The discovery of piRNA pathway well explains some long-lasting evolutionary observations*,* such as the *I-R* system [25], [26] and the *P-M* system that induce hybrid dysgenesis in *Drosophila* [27], [28], [29], [30]. Here we briefly summarize the research progress in the biogenesis, mechanisms, and functions of piRNAs in the model organism *D. melanogaster*, and then we discuss the possible evolutionary implications for the interactions between TEs and piRNAs.

## TEs

The content of TEs varies widely in eukaryotic genomes, ranging from 1% [31] to 80% [32]. According to the mechanisms of mobilization, TEs are classified as transposons and retrotransposons [13]. A transposon is a moveable DNA fragment in the genome that can be transposed to another location by “cut and paste”, while a retrotransposon is inserted into new locations by reverse transcription of RNA intermediates and replicates in the manner of “copy and paste” [13]. TEs can even be passed between species through horizontal transfer [33].

During long-term evolution, TEs have shaped the composition of the host genome through a variety of mechanisms [34], [35], [36], [37], [38]. First, copies of homologous TEs scattered across the genome can induce ectopic recombination [39], [40], [41]. Second, TEs could be domesticated into new domains of protein-coding genes [42], [43], [44], [45]. Third, exaptation of TEs can provide new motifs to regulate gene expression [46], [47], [48]. TE insertions have been nicely demonstrated to contribute to the adaptive evolution of the host organisms by influencing expression of nearby genes [49], [50], [51], [52]. Although TEs greatly influence the evolution of the genomes, they are generally detrimental to the host organisms because of: (1) destructing coding and regulatory regions of genes [53], [54], [55], [56], [57]; (2) depleting cellular energy and resources [58], [59]; or (3) mediating deleterious chromosomal rearrangements through ectopic recombination [39], [60], [61], [62].

TEs have been extensively studied in *Drosophila* for decades [63], [64], [65]. About 120 TE families have been identified in *D. melanogaster* [66], which make up at least 5% of the euchromatic genome [66], [67], [68], [69], [70], [71]. There are ∼2000 inactive copies of the inactivation escape 1 (*INE-1*) gene family in *D. melanogaster*, although the copy number ranges from 1 to ∼300 for most TE families [72]. Based on the expression patterns, TEs could be classified into germline-specific, somatic, and intermediate groups [73], [74]. Most TEs in *Drosophila* are active, and approximately 50%–80% of the mutations that generate observable phenotypes in *D. melanogaster* are attributable to TEs [54], [75]. It is estimated that the TEs decrease the fitness of *D. melanogaster* by 0.4%–5% [62], [76], [77], [78].

## Argonaute proteins

The repertoire of sRNAs has been expanding since the RNA interference (RNAi) mechanism was discovered [79], [80]. Argonaute (AGO) proteins bind to sRNAs and form the RNA-induced silencing complex (RISC), in which sRNAs recognize the target genes with complementary sequences and AGO proteins cleave and repress the targets. AGO proteins consist of four domains including the N-terminal domain, a PAZ domain that binds to RNAs, a MID domain that binds to the cap structure of mRNA, and a PIWI domain that is essential for target cleavage [81]. AGO proteins are ancient and can be found in nearly all eukaryotes except *Saccharomyces cerevisiae* [82], [83]*.* The size of AGO family varies across species, with eight genes present in mammals [84], five in flies [23], and 27 in worms [85]. The AGO proteins are divided into three clades including AGO, PIWI, and worm-specific AGO (WAGO) clades [82]. Both the microRNAs (miRNAs) and small interfering RNAs (siRNAs) bind to AGO proteins and are involved in posttranscriptional gene silencing process in cytoplasm [81], [86], whereas PIWI proteins are predominantly expressed in gonads and bind to piRNAs to silence TEs [87], [88]. WAGO proteins are involved in the unique RNAi system in nematodes [85].

*Drosophila* genomes contain five AGO genes, including two members from AGO clade (*ago1* and *ago2*) and three members from PIWI clade (*piwi*, *aub*, and *ago3*). The localization of the three PIWI proteins is different. Aub and Ago3 are located at the perinuclear electron-dense nuage in the germline cells, while the predominant localization of Piwi is the nucleus in both germline and somatic cells of ovary [23]. Piwi is also located in the cytoplasm during the development of eggs [23]. These three PIWI proteins show strand preference in binding to piRNAs. piRNAs antisense to TE transcripts are mainly bound by Piwi and Aub, whereas piRNAs sense to TEs are predominantly bound by Ago3 [23]. Since Drosha and Dicer are not involved in piRNA machinery, the biogenesis pathway of piRNAs is different from miRNAs and endogenous siRNAs [79], [89]. piRNA biogenesis is very complicated and the detailed mechanisms need further elucidation, but it is well established that Piwi, Aub and Ago3 participate in piRNA biogenesis and target silencing through a “Ping-Pong” model [23], [90].

## piRNAs in *Drosophila*: a snapshot

In *Drosophila*, piRNAs are 23–29-nt sRNAs mainly expressed in germline cells [23]. The first identified piRNAs were repeat-associated siRNAs (rasiRNAs) from the Y-linked *Suppressor of Stellate* locus in *D. melanogaster* [16]. These piRNAs were uncovered to silence the X-linked tandem-repeated *stellate* genes [16], [91]. Later on, piRNAs were discovered as master regulators to repress TEs in *Drosophila* and other model organisms such as mice, rats, nematodes, and zebrafish [17], [18], [19], [20], [21], [22], [23], [24]. The piRNA repertoire is very complex, with thousands of distinct piRNA sequences present in the genomes of *Drosophila* [23]. Moreover, there is no structural or sequence similarity between the sequences of different piRNAs, except for a strong bias for uracil in the first nucleotide [23]. In *Drosophila*, piRNAs recognize their targets, which are mainly mRNAs of active TEs, through perfect or nearly perfect antisense matching. piRNAs repress their target mRNAs through the “Ping-Pong” cycle [23], [92], [93], [94], [95], [96].

## piRNA clusters

Genome-wide mapping of piRNAs revealed that most piRNAs in *D. melanogaster* were derived from discrete loci, also known as piRNA clusters [23]. Only a small fraction of piRNAs were generated from genic regions, such as from the 3′ UTR of *tj* [97], [98]. At least 142 piRNA clusters were defined in the genome of *D. melanogaster* and these clusters are enriched in repetitive sequences or inactive TE fragments [23], [74], [99], [100]. The piRNA clusters range up to 200 kb, and they are preferentially located in the heterochromatin regions [23] characterized by the marks of trimethylation at lysine 9 of histone H3 (H3K9me3) bound by heterochromatin protein 1 (HP1) [101], [102], [103], [104], [105], [106]. These heterochromatic regions usually have low recombination rates and hence reduced efficiency of purifying selection, which putatively serve as “safe harbors” for TEs to accumulate and to develop into piRNA clusters [107]. Furthermore, it has been neatly demonstrated that heterochromatin formation is important for the proper production of piRNAs [102], [103], [104], [105], [106].

Based on the strand distribution of mature piRNAs, piRNA clusters are classified into “uni-strand” and “dual-strand” clusters. The “uni-strand” clusters have piRNAs mapped onto one genomic strand, such as the *flamenco* cluster located on the X chromosome and extending over 180 kb, which is responsible for the somatic piRNAs in the follicle cells (the somatic cells surrounding germline cells) in gonads [23]. The “uni-strand” piRNA cluster might be transcribed by canonical RNA polymerase II (RNAPII) [93], [108]. For example, the *flamenco* cluster is activated by the cubitus interruptus (Ci) protein and the precursor transcript of *flamenco* undergoes alternative splicing to generate diverse piRNA precursors [108]. It is of note that one *P* element inserted at the 5′ end of the *flamenco* cluster results in the failure of all transcripts on this cluster [23], [74].

The “dual-strand” clusters, which give rise to most piRNAs in the germlines of *D. melanogaster*, generate piRNAs that are mapped onto both strands [23]. The “dual-strand” clusters generally do not exhibit signatures of RNAPII transcription since they lack clear promoters, 5′ methyl-guanosine caps, and clear transcription termination signals [93]. It was proposed that the heterochromatin protein Rhino, Deadlock, and the transcription termination cofactor Cutoff form the “RDC” complex, which mediates transcription of dual-strand piRNA clusters in *Drosophila* ovaries [93], [94]. Moreover, Rhino, Cutoff, and RNA helicase UAP56 are required to inhibit the splicing of the precursor transcripts for piRNAs [94]. Furthermore, transcription of both strands of a piRNA cluster is required for proper production of piRNAs [94].

## Maturation of primary piRNAs

**Figure 1:**
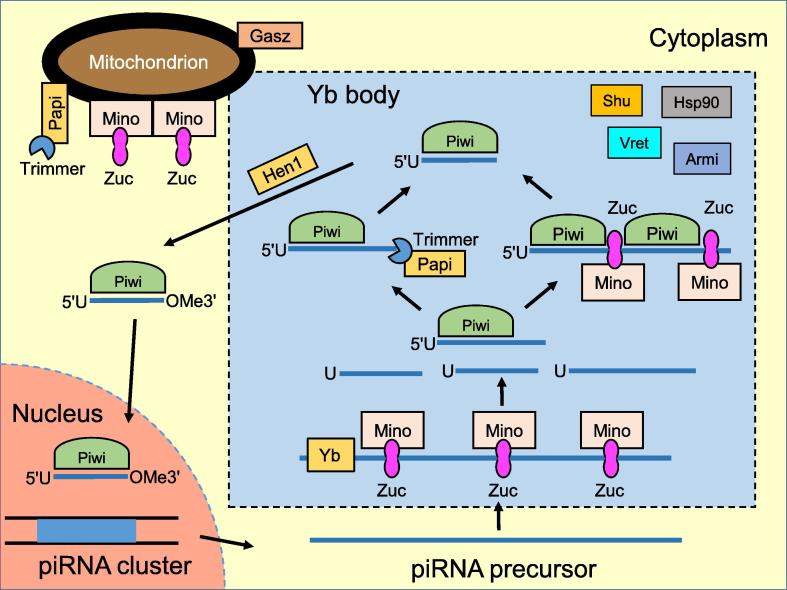
**The primary piRNA biogenesis in ovarian somatic cells of *D. melanogaster*** The long piRNA precursor is transcribed from a piRNA cluster in nucleus, and then transported into cytoplasm and recruited into Yb body. The piRNA precursor is cleaved by Zuc and its cofactors, producing piRNA intermediates with uracil (U) at the 5′ end. After Piwi being loaded onto piRNA intermediates from the 5′ end, the 3′ end of piRNA intermediate could be produced either by the exonuclease Trimmer and its cofactor Papi, or another cleavage by Zuc with the generation of Piwi-bound phased piRNAs. After 2′-*O* methylation (OMe) at the 3′ end by Hen1 in the Yb body, the mature piRNA complexes enter the nucleus to silence genes at the transcriptional level. Other proteins involved in this process include Yb, Mino, Armi, Vret, Shu, Hsp90, and Gasz. PIWI, *P*-element-induced wimpy testis; piRNA, PIWI-interacting RNA; Zuc, Zucchini; Mino, Minotaur; Armi, Armitage; Vret, Vreteno; Shu, Shutdown; Hsp90, heat shock protein 90; Papi, partner of PIWI. This figure is drawn based on [90] and relevant literature.

In follicle cells, only Piwi, but not Aub or Ago3, is expressed [23]. The primary piRNA biogenesis is shown in Figure 1. The transcript of uni-strand piRNA cluster is first transported into the Yb body of the cytoplasm [109]. Zucchini (Zuc), which is located on mitochondrial outer membrane, cleaves the long single-stranded transcript and generates the piRNA intermediates [110], [111]. Then the 5′ end of piRNA intermediates is loaded on Piwi in the Yb body of follicle cells [109]. The observed strong bias for 5′ uracil (termed 1U bias) of piRNAs is demonstrated to be related to the MID domain of Piwi [112]. Next, piRNA 3′ end is trimmed to the mature length by Nibbler (Nbr) [113], [114] or Trimmer and its cofactor partner of PIWIs (Papi) [115], [116]. When the 3′ trimming stops at the piRNA intermediate region that is protected by Piwi, the 3′ end is methylated by Hen1 and forms a 2′-*O*-methylated (OMe) modification of the mature primary piRNA [117], [118]. In follicle cells, the mature primary piRNAs are able to recognize and destroy target transcripts in a posttranscriptional manner [74]. Many other proteins also participate in the primary piRNA maturation in the follicle cells. These include the Tudor protein Yb [119], [120], Vreteno (Vret) [109], [121], Minotaur (Mino) [122], [123], Gasz [124], helicase Armitage (Armi) [125], chaperone Shutdown (Shu) [126], and heat shock protein 90 (Hsp90) [127], many of which are anchored in the outer membrane of mitochondria.

**Figure 2:**
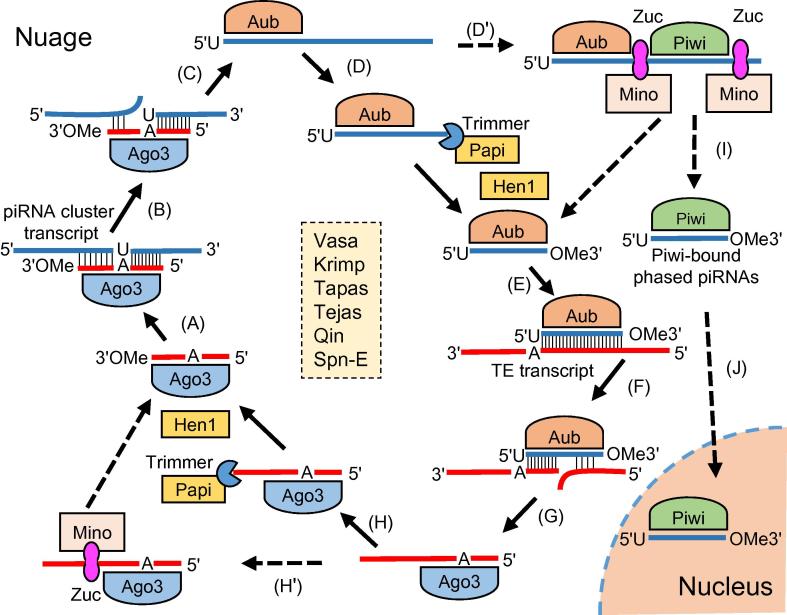
**The “Ping-Pong” cycle amplifies piRNAs and silence TEs** The Ago3-bound sense piRNA binds to complementary sequence on piRNA cluster transcript (**A**). The piRNA cluster transcript is cleaved by Ago3 and the 5′ end with uracil (U) of the antisense piRNAs is formed (**B**). Aub loads onto the antisense piRNA intermediates (**C**). Then the 3′ end of piRNA is trimmed to the mature length either by exonuclease Trimmer and its cofactor Papi (**D**) or through cleavage by Zuc with the generation of Aub-bound piRNAs and Piwi-bound phased piRNAs (**D’**). After 2′-*O* methylation (OMe) by Hen1, the mature Aub-bound piRNA binds to the TE transcript (**E**). The TE transcript is then cleaved, and the 5′ end of the sense piRNA is generated with A in the 10th position (**F**). Ago3 loads onto the sense piRNA intermediates (**G**), and the 3′ end of piRNA is trimmed to the mature length either by exonuclease Trimmer and its cofactor Papi (**H**) or through cleavage by Zuc (**H’**). After 2′-*O* methylation by Hen1, the mature Ago3-bound piRNA is generated and used to maintain the cycle. The Piwi-bound phased primary piRNAs are processed into mature phased piRNAs with methylation in the 3′ end (**I**). The phased piRNAs enter into the nucleus (**J**). The proteins required for the “Ping-Pong” cycle are listed in the center, including Vasa, Krimp, Tapas, Tejas, Qin, and Spn-E. PIWI, *P*-element-induced wimpy testis; piRNA, PIWI-interacting RNA; Ago3, Argonaute 3; Aub, Aubergine; Zuc, Zucchini; Krimp, Krimper; Spn-E, Spindle-E; Papi, partner of PIWI. This figure is drawn based on [90] and relevant literature.

In germline cells, Vasa and UAP56 recognize the transcripts of piRNA clusters and transport them from nucleus into nuage in the cytoplasm [128]. The primary piRNA processing in germline cells is similar to that in follicle cells, except that Piwi, Aub, and Ago3 are all expressed, but only Piwi and Aub load the mature primary piRNAs. Aub-bound primary piRNAs, together with Ago3, generate secondary piRNAs through a “Ping-Pong” cycle (Figure 2). The production of primary piRNAs requires many effectors such as Armi, Zuc, Shu, and Hsp83. The 2′-*O*-methylation is finally formed at the 3′ end of a primary piRNA by Hen1 [126], [129], [130]. Interestingly, in both follicle and germline cells, the 3′ end formation of primary piRNA is either trimmed as described above, or further cleaved by Zuc to produce the Piwi-bound phased piRNAs [95], [96] (Figure 1, Figure 2).

## The “Ping-Pong” cycle amplifies secondary piRNAs and silences targets

Unlike the primary piRNA pathway, the “Ping-Pong” cycle generating the secondary piRNAs is restricted to the electron-dense nuage of *Drosophila* germline cells [23]. The “Ping-Pong” cycle (shown in Figure 2) is a well-established mechanism in TE suppression in germ cells (reviewed in [87], [88], [90], [131], [132], [133], [134]). Briefly, in the “Ping-Pong” cycle, an Ago3-associated piRNA recognizes a complementary transcript (usually from an active TE) and Ago3 cleaves the target at the site corresponding to the 10th nucleotide of the Ago3-bound piRNA, thereby generating a new piRNA loaded by Aub. Then the Aub-loaded piRNA in turn recognizes and cleaves a complementary TE transcript, generating a new piRNA identical to the initial Ago3-loaded piRNA. The piRNAs are amplified during these “Ping-Pong” cycles, leaving a 10-bp region overlapping between the sense and antisense piRNAs [23], [74]. The “Ping-Pong” model also consumes transcripts of TEs and thus silences TEs. Several piRNA pathway-associated factors have been demonstrated to participate in this process, such as Spindle-E (Spn-E) [74], Krimper (Krimp) [135], [136], Tejas [137], Tapas [138], Vasa [128], [139], and Qin [140], [141]. Notably, Piwi does not directly participate in the “Ping-Pong” cycle. The Piwi/piRNAs complex is imported into the nucleus to repress TEs by modifying chromatin [104], [105], [142], [143], and Panoramix (Panx) is required in the piRNA-driven recognition of transposons to silence their transcription [144].

The “Ping-Pong” cycle only amplifies the primary piRNAs and is thus not adequate to account for the extraordinary diversity of piRNAs observed in the germline cells [145]. During the “Ping-Pong” cycle, Aub-associated piRNA intermediates can also be cleaved by Zuc in the downstream transcript regions, generating phased piRNAs that are bound by Piwi [95], [96]. The spreading mechanism of phased piRNAs well explains the extremely high diversity of piRNA repertoire and provides the host versatility to defend against various invasive TEs.

## Hybrid dysgenesis caused by interactions between TEs and piRNAs

The discovery of piRNA pathway elucidates the molecular mechanisms underlying hybrid dysgenesis for the *P-M* system [11], [28], [29], [30], [33] and the *I-R* [25], [26], [29] system.

**Figure 3:**
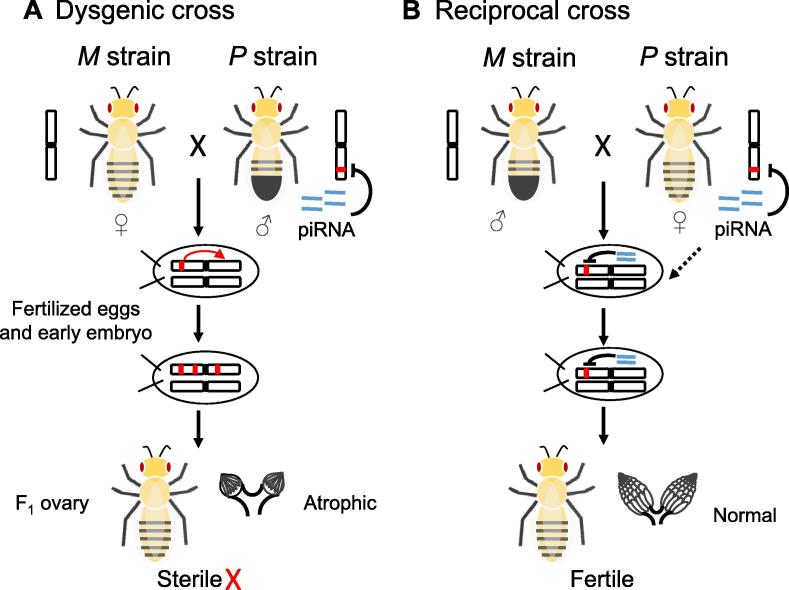
**Hybrid dysgenesis induced by the interactions between piRNAs and *P* element in *D. melanogaster*** **A.** Dysgenic cross: the crossing between *M* females (without *P* element) and *P* males (with *P* element) produces sterile offspring since the active transposition of *P* element disrupts genome and induces gonadal atrophy. **B.** Reciprocal cross: the crossing between *M* males and *P* females produces fertile offspring since the maternally inherited piRNAs repress activities of *P* elements in the offspring. piRNA, *P*-element-induced wimpy testis (PIWI)-interacting RNA.

*P* element is a DNA transposon that is transcribed in both soma and germline, whereas active transposase is only translated in germline cells [146]. The complete sequence of *P* element is 2.9 kb in length. Most *P* elements have internal deletions and do not encode transposase, thus relying on the complete *P* elements to transpose [146]. Some strains of *D. melanogaster* do not carry any *P* element (*M* strains), while other strains have multiple *P* elements in the genomes (*P* strains). When *P* males and *M* females are mated, the F_1_ offspring usually suffer from the syndromes of hybrid dysgenesis, such as recombination in male flies, higher mutation rates, dysgenic gonads, and frequent sterility [11], [147]. However, the hybridization between *P* females and *M* males, between *M* females and *M* males, or between *P* females and *P* males, does not generate hybrid dysgenesis. The mechanism for such a puzzling observation got fully appreciated after the piRNA pathway was discovered (Figure 3). In the *P* strains, piRNAs were developed to specifically repress *P* elements [29]. *P* element-corresponding piRNAs are maternally deposited in the F_1_ embryos by *P* females, therefore *P* elements, if present, are repressed in the embryos. However, in embryos from the crossing of *M* females and *P* males, there are no piRNAs repressing *P* elements, therefore *P* elements actively transpose and disrupt genes crucial for the normal development of the F_1_ offspring [29].

The functional *I* element is a non-long terminal repeat (non-LTR) retrotransposon that is 5.4 kb in length in *Drosophila. I* element is specifically transcribed in female germline cells of *Drosophila* [25], [26], [148]. The functional *I* elements are polymorphic in *D. melanogaster*. The *I* (inducer) strains have functional *I* elements, whereas the *R* (reactive) strains lack functional *I* elements. *I* elements are very active in F_1_ females (called SF females, which are usually sterile) from the crossing of *R* females and *I* males. Nonetheless, when *I* females and *R* males are mated, *I* elements are less active in F_1_ females (called RSF females) [25], [26], [148]. It is also shown that in the *I* strains, piRNAs specifically repress *I* elements through the “Ping-Pong” cycle and the maternal *I-*specific piRNA deposition is important for silencing functional *I* elements in the F_1_ adults. In contrast, lack of maternal *I*-specific piRNAs in the F_1_ adults from the crossing of *R* females and *I* males would cause hybrid dysgenesis [29].

Taken together, the maternal deposition of TE-specific piRNAs neatly explains the reciprocal cross difference in hybrid dysgenesis between *P* and *M* strains, or between the *I* and *R* strains of *D. melanogaster* [29].

## *De novo* piRNAs induced by TE insertions

How piRNAs are developed to repress a novel invasive TE is not well understood at this moment. However, several studies have demonstrated that *de novo* piRNAs could be rapidly produced after invasions of novel TEs. For example, *P* elements invaded *D. melanogaster* by horizontal transfer from *Drosophila willistoni* within the last 100 years, and they remain polymorphic in the populations of *D. melanogaster* [28], [33]. Notably, abundant piRNAs specifically repressing *P* elements are readily detected in the *P* strains [29]. Furthermore, the adaptation to the novel *P* element insertion could even occur within the lifetime of a single fly [30]. *Penelope* is a member of the *Penelope*-like element (PLE) family and it remains polymorphic in *Drosophila virilis* [149]. After artificially transforming intact *Penelope* into *D. melanogaster*, piRNAs (mainly in ovary) and siRNAs (mainly in somatic cells) that specifically repressed *Penelope* transcripts were frequently detected in the transgenic strains even after 10 years [142]. Furthermore, some intact *Penelope* copies are inserted into pre-existing piRNA cluster of *D. melanogaster*, suggesting invasive TEs could be trapped by the established piRNA clusters to silence themselves and to generate piRNAs to repress the homologous TEs as well [107].

Other transgenic experiments demonstrate that TEs inserted outside of the pre-existing piRNA clusters can also trigger the production of *de novo* piRNAs. Such piRNAs were detected in the inserted TEs and the flanking regions that were bi-directionally transcribed [150]. Further analysis demonstrate that in *Drosophila* gonads, a considerable amount of TE insertions triggered the production of *de novo* piRNAs that are mapped on both strands of the inserted TEs and flanking regions [151], [152]. Novel insertions of TEs would induce H3K9me3 modifications that are required for the production of *de novo* piRNAs around the insertion sites [150], [153].

## Interactions between TEs and piRNAs at the population level

The polymorphism of TE insertions among individuals of *D. melanogaster* provides a model system to investigate TE evolution at the population level [39], [53], [60], [61], [62]. Although TEs are deleterious and under strong negative selection, they reproduce fast in the genomes so that they persist in the populations. The traditional “transposition-selection” population genetics model assumes that the equilibrium between TE replication and purifying selection determines TE abundance in the genome [53], [62]. By investigating patterns of TE insertion polymorphism in five populations from North American and one population from sub-Saharan Africa in *D. melanogaster*, it is shown that TEs are subject to purifying selection due to ectopic recombination [154]. The intensity of purifying selection varies with recombination rate of the inserted region, virulence of individual TEs, and the natural history of the TE families [154]. Similar results have also been reported in a Portugal population [155] and in an American population from the *Drosophila* Genetic Reference Panel (DGRP) [156].

However, for most population genomic analysis of TEs, the effect of piRNA repression has not been considered. Therefore, it remains unclear whether or not the possible arms-race process between TEs and piRNAs would affect the landscapes of TE insertions. For example, the *flamenco* locus has been known to regulate the retrotransposons *Gypsy* [157], [158], *ZAM*, and *Idefix* [159] for a long time, and the repression efficiency differs across strains [159]. 79% of all the piRNAs matching *ZAM* are produced by *flamenco* cluster, while 30% of all the piRNAs targeting *Idefix* and 33% targeting *Gypsy* are also from *flamenco* [23]. Although this piRNA cluster is ancient and generates primary piRNAs in both *D. melanogaster* and *D. erecta* [74], the fine-scale structure and composition of *flamenco* are quite different among three strains of *D. melanogaster*, which well explains their difference in TE repression [160].

**Figure 4:**
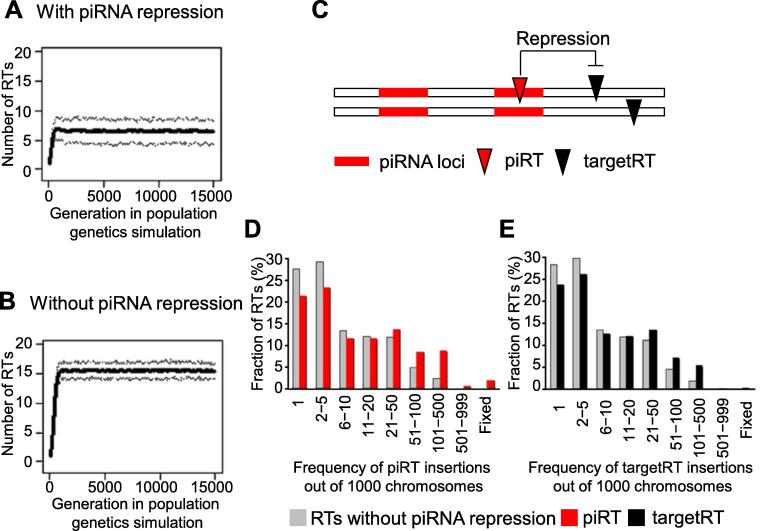
**The interactions between piRNAs and TEs in the framework of population genetics** With the same parameter settings in the population genetics simulations, the number of TEs in one chromosome is significantly lower in the presence of piRNA repression (**A**) compared to the scenario that piRNAs do not repress TEs (**B**). The solid line represents the mean number of RTs in each chromosome, whereas the thin dashed lines represent the confidence intervals of 90%. **C.** A schematic illustration of the interaction between piRNAs and TEs. piRTs (RTs in the piRNA clusters, shown in red inverted triangle) refer to the RT that jump into piRNA loci and generate piRNAs to repress RTs of the same family, whereas targetRTs are RTs present outside of piRNA loci, whose activity is reduced by the expressed piRNAs. **D.** piRTs increase the fitness of the hosts. They are driven by positive selection and spread in the population rapidly, which is manifested by their higher frequencies in the population. **E.** The frequency of the targetRTs is also skewed toward higher frequencies because their deleterious effects are alleviated by the repression of piRNAs. The figures are adapted from [107]. piRNA, *P*-element-induced wimpy testis (PIWI)-interacting RNA; RT, retrotransposon; TE, transposable element.

Lu and Clark were among the first to incorporate piRNA emergence and repression into the population genetic framework of TEs [107]. By combining extensive evolutionary modelings and empirical TE polymorphism analysis, they showed that piRNAs significantly reduced the fitness costs of TEs and that the novel insertions generating piRNAs are favored by natural selection (Figure 4). Such piRNA-generating TE insertions will spread quickly in the populations or even reach fixation (Figure 4). However, the piRNA repression also provides a shelter for the TEs to accumulate, since the deleterious effects of TEs are alleviated in the presence of piRNAs. The fitness of the host depends on the continuous repression of TEs by piRNAs, and piRNA maintenance is therefore very important to the host organism [107]. Further studies indicate that the interactions between TEs and piRNAs are very complicated. It seems that there is no simple relationship between the copy number of TEs and the abundance of piRNAs [152], [161].

## Adaptive evolution of piRNA machinery

The effector proteins in piRNA pathway are important in piRNA generation and TE silencing. Many effector proteins exhibit signatures of adaptive evolution [162], [163], [164], [165]. In *Drosophila*, Lee and Langley found more evidence of adaptive evolution for these effector genes than for the pathogen-interacting immunity genes [166]. It was thus proposed that the changes in TE abundance between species caused the rapid evolution of piRNA pathway genes [167]. The arms race between TEs and the piRNA effector proteins would potentially cause the species with more TEs to have a higher rate of evolution in the amino acid sequences of piRNA effector proteins. However, such a model was not supported by empirical data [168]. Furthermore, the expression levels of piRNA pathway genes do not have correlations with novel TE abundance across strains in *D. melanogaster* [152] or in *Drosophila simulans* [169]. Taken together, the molecular mechanism underlying the adaptive evolution of the piRNA machinery remains a mystery. New models and further investigations are required.

## Hsp90: piRNA pathway effector or canalization capacitor?

**Figure 5:**
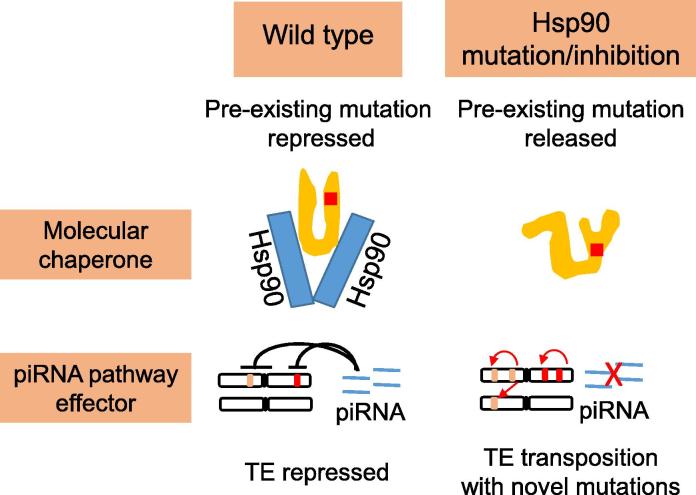
**The possible role of Hsp90 in canalization *vs.* TE suppression** In the canalization model, Hsp90 might help the client proteins to fold correctly, even though they may carry deleterious mutations that would otherwise destabilize these proteins in the absence of Hsp90. In other words, Hsp90 masks the deleterious effects of the mutations. Abolition of Hsp90 releases the deleterious effects of the pre-existing mutations, thus resulting in the manifested phenotypes. In the piRNA pathway effector model, Hsp90 is involved in piRNA biogenesis. Hsp90 mutation results in active TE transposition, thus generating new mutations and abnormal phenotypes. piRNA, *P*-element-induced wimpy testis (PIWI)-interacting RNA; TE, transposable element; Hsp90, heat shock protein 90.

Hsp90 is involved in piRNA biogenesis in that it facilitates precursor piRNAs loading onto PIWI proteins accurately [127]. These discoveries call into question that Hsp90 functions as a canalization capacitor [170]. “Canalization” was coined by Waddington to describe the ability that organisms evolve to stabilize the phenotypes against genetic and environmental perturbations [171]. It was postulated that Hsp90, a chaperone and heat-shock protein, functions as a canalization capacitor to mask the deleterious effects of many pre-existing “cryptic variation” [170] (shown in Figure 5). This hypothesis was supported by the observations that there were many morphological abnormalities in the fly mutant of *Hsp83*, which encodes Hsp90 [170]. This hypothesis attracts tremendous interest from biologists, especially from evolutionary biologists, since it provides a new framework to investigate how genetic diversity is maintained. However, it was later demonstrated that Hsp90 was involved in biogenesis of piRNAs in *Drosophila*, and mutation of *Hsp83* failed to suppress novel mutations caused by the active TEs in the germline cells [172]. In other words, the phenotypic abnormalities observed in the *Hsp83* mutants are more likely to be caused by novel mutations due to TE insertions rather than the release of pre-existing cryptic mutations. Therefore, the hypothesis that Hsp90 functions as a canalization capacitor should be reconsidered [173].

## Conclusions and perspectives

The genomes, transcriptomes, and proteomes of the host organisms have been greatly shaped by the genomic conflict between TEs and the host genome. Based on the theoretical and experimental studies from TE polymorphism in *D. melanogaster*, the traditional “transposition-selection” population genetics model assumed that the TE abundance in the genome is determined by the equilibrium between TE replication and purifying selection. Over the last decades, the discovery of piRNAs has revolutionized our understanding of the molecular mechanisms in TE repression. The interaction between piRNAs and TEs well explains the hybrid dysgenesis for the *I-R* and *P-M* systems in *Drosophila.* The population genetic analysis also shows the importance of piRNA maintenance to the host organism.

However, several fundamental questions remain to be further investigated. First, what are the major mechanisms for the *Drosophila* hosts to adapt to novel TE insertions? Many detailed questions need to be explored. How soon are piRNAs generated after the invasion of TEs? How did piRNA clusters origin and evolve? What are the molecular mechanisms underlying the rapid evolution of the piRNA machinery? What are the major forces governing such an evolutionary process? Second, how does piRNA repression, coupled with natural selection, shape the landscape of TE insertions? What is the relative importance of piRNA pathway in preventing TE accumulation compared to other mechanisms such as purifying selection due to ectopic recombination? Third, what are the consequences of the arms-race between TEs and piRNAs in the host genome? Given the large and complex repertoire of piRNAs, how are they regulated to avoid interfering with the normal transcriptomes of the germline cells? How frequently can we observe the arms-race processes between TEs and piRNAs? Do the interactions between piRNAs and TEs cause widespread weak hybrid dysgenesis that leads to the population differentiation? More investigations are needed to address these questions. Answers to these questions will undoubtedly help us better understand some fundamental questions in evolutionary biology.

## Competing interests

The authors have declared that no competing interests exist.
